# International regulatory and publicly-funded initiatives to advance drug repurposing

**DOI:** 10.3389/fmed.2024.1387517

**Published:** 2024-05-31

**Authors:** Eva Louise Spin, Aukje K. Mantel-Teeuwisse, Anna Maria Gerdina Pasmooij

**Affiliations:** ^1^Dutch Medicines Evaluation Board, Utrecht, Netherlands; ^2^Division of Pharmacoepidemiology and Clinical Pharmacology, Utrecht Institute for Pharmaceutical Sciences (UIPS), Utrecht University, Utrecht, Netherlands

**Keywords:** drug repurposing, drug repositioning, drug development, regulatory agencies, regulatory science

## Abstract

**Introduction:**

Although drug repurposing holds great potential in addressing unmet needs, successful practical implementation is challenging and has been less widespread than anticipated. Regulators may play a critical role in addressing this, and recent years have seen the conception of regulator-initiated and publicly-funded repurposing initiatives, with significant regulator involvement.

**Methods:**

International regulators and public funders (*n* = 8) were interviewed to obtain insight in how repurposing can be advanced from a regulatory perspective. Transcripts were thematically analyzed.

**Results:**

Most initiatives employed a broad concept of repurposing. While patient access was the main focus, label extension remained the gold standard. Commonly perceived barriers were a lack of regulatory expertise, limited downstream drug development, insufficient financial incentives, inadequate awareness of challenges, and poor collaboration. Ways for regulators to facilitate repurposing include early and accessible involvement fostering education, collaboration, and awareness. Increased stakeholder engagement, including internationally, was recommended. Legislative changes may enable the current repurposing ecosystem to evolve.

**Discussion:**

Regulators may play a central role in advancing repurposing by reconsidering their responsibilities within the current regulatory framework, both in mitigating repurposing pitfalls and actively encouraging repurposing initiatives by industry and non-traditional drug developers.

## 1 Introduction

Drug repurposing has increasingly been a focus of the drug developing ecosystem, ostensibly gaining traction since Ashburn & Thor’s landmark publication in 2004 (1). Drug repurposing can be defined as the identification, development, and/or use of an existing active pharmaceutical ingredient (API) or medicinal product for a new disease, patient population, dosage form, duration, route of administration, or combination of the aforementioned (2). From the most influential drugs in terms of revenue (sildenafil) and notoriety (thalidomide) (3), to unprecedented global urgency during the COVID-19 pandemic (dexamethasone, tocilizumab, and baricitinib in the UK RECOVERY trial) (4), prior examples illustrate the wealth of possibilities and untapped potential of repurposing.

Repurposing’s societal and scientific relevance stems from its potential to address unmet medical needs (e.g., rare or sporadic infections, emerging threats, rare cancers) in a safe and timely manner at a lower cost by leveraging existing knowledge of repurposing candidates. Examples include the use of propranolol in infantile hemangioma, and fenfluramine in treating the rare Dravet syndrome and Lennox–Gastaut syndrome (5).

While past decades have seen exceptional scientific and technological progress, pharmaceutical research and development (R&D) costs have increased exponentially without proportionate public health advancement (1, 6). Compounds that have already been established as safe during preclinical or human studies may present a de-risked approach to the notoriously failure-prone drug development process and “the valley of death,” in which only 0.1% of experimental entities proceed from preclinical to human testing (6). For drugs that have advanced to phase I clinical trials, an estimated 9 out of 10 candidates fail during clinical assessment and approval (7). For repurposed drugs, the success rate is thought to be higher (8), although a full overview of success rates in repurposing does not yet exist Repurposing may expedite the drug development process from initial concept to market access from an average 10–17 years to 3–12 years, through the use of previously established knowledge of repurposing candidates (1). Besides safety assessments, this may include known pharmacokinetic profiles, formulation development, and regulatory knowledge (3, 9). In some cases it may be possible to rely on prior data (e.g., target and compound discovery and screening, lead optimization, absorption, distribution, metabolism, excretion, toxicity (ADMET) assessment, phase I and II trials) for approval of a new indication, allowing for a more rapid progression to late-phase clinical trials, and ultimately patient access (1). For instance, the IRDiRC Drug Repurposing Guidebook provides ample starting points on how to optimize repurposing processes, highlighting which steps are of vital importance in doing so (e.g., patent status) (10). In short, repurposing may be safer and quicker than *de novo* drug development, at reduced costs. Given rapidly increasing health care costs, this presents a major advantage (11). The estimated total costs of bringing a repurposed drug to market are thought to be 50–60% of R&D costs of *de novo* drug development (12).

Repurposing’s advantages seem tantalizingly straightforward. However, its practical implementation has proven to be less widespread than initially hoped for (13). Major hurdles to repurposing were highlighted by Austin et al. in 2021, as discussed at the National Center for Advancing Translational Sciences (NCATS), Reagan-Udall Foundation (RUF) and FDA workshop on repurposing in 2019 (14). These include lack of centralization of information on repurposing resources, poor data access, barriers to clinical utilization of repurposed drugs (including on- versus off-label use), and lack of financial and regulatory incentives.

Of these barriers, regulatory challenges may present a distinct opportunity for improvement by addressing the cost in time and money of repurposing. Examples of regulatory hurdles include, but are not limited to, the following aspects. The current regulatory and legal framework offers little to no incentive for companies or other drug sponsors to pursue repurposing when it comes to off-patent drugs (9). Notwithstanding the aforementioned R&D savings, repurposing still requires substantial investments in terms of risk, costs, and workload. Without traditional exclusivity models and in the face of generic competition, it can be difficult for sponsors to secure a return on these investments (15). Reluctant industry engagement may present a challenge even if initial research efforts are led by other, non-commercial stakeholders, since, ultimately, only the marketing authorization holder (MAH) can apply for an original marketing authorization (MA) to be adjusted. For academia and not-for-profit developers, an important barrier may be lack of regulatory knowledge and experience. This is especially significant given that the majority of repurposing initiatives originate in academia, while the skills and resources to facilitate translational development traditionally lie with industry (16). This currently means that the majority of academia-initiated drug innovation never progresses beyond early development (17). To mitigate these and other issues, increased regulatory involvement and collaboration is often recommended in literature, by regulators and drug developers alike (18). By accommodating the aforementioned concerns, regulators can play a critical role in advancing drug repurposing as a whole (9). This call to action has not gone unnoticed, and recent years have seen the conception of several projects to address these challenges. Examples are the European Medicines Agency (EMA) pilot on repurposing of authorized medicines (19), the International Rare Diseases Research Consortium (IRDiRC) Drug Repurposing Guidebook (10), and the FDA and National Institutes of Health (NIH) joint initiative CURE ID, which focusses on data collection of real-world data on repurposed drugs (20).

An overview of existing regulator and publicly-funded initiatives, with significant regulator involvement, to stimulate and facilitate repurposing (referred to as ‘repurposing initiatives’ in this study) is lacking. Insight into the current regulatory landscape is critical in determining regulators’ current position on repurposing, and what can be learnt from existing initiatives. This study aimed to answer how drug repurposing can be advanced from a regulatory perspective, based on current regulator and publicly-funded repurposing initiatives.

## 2 Methods

A series of semi-structured interviews with international regulators and leaders of publicly-funded repurposing efforts was at the core of this study, and enabled a more in-depth investigation of identified initiatives and perceived regulatory considerations and challenges identified through a literature review-based landscape analysis of repurposing initiatives.

### 2.1 Literature review

Current literature and publicly accessible online resources were reviewed in order to create a landscape analysis of repurposing initiatives. PubMed, Google Scholar, and public websites for regulatory agencies and publicly-funded institutions were consulted between January 23th and March 3th 2023 for appropriate information using (a combination of) search terms, covering “drug repurposing, repositioning, reprofiling, redirecting, rediscovery,” “regulatory, regulator,” and the respective agencies’ names. Sources were selected based on relevancy (e.g., relating to the regulatory aspects of drug repurposing), reliability (e.g., peer reviewed) and recency, as relating to the main research question (articles published after 2003, as Ashburn and Thor published their landmark study in 2004). Search results were complemented by background literature on repurposing. These findings provided the basis for part II of this study.

### 2.2 Interviews

Eligible interview candidates were international regulators and leaders of publicly-funded repurposing initiatives, with significant regulator involvement, identified through purposive and snowball sampling resulting in a literature review-based landscape analysis of repurposing initiatives. Eligibility criteria for initiatives were: to be ongoing at the time of analysis (spring of 2023), to entail active involvement of one or more regulators in an official capacity, and to include multiple repurposing cases (e.g., not a project focused on the repurposing of a single drug).

Interview questions covered:

1. initiative characteristics—aims, definition of ‘repurposing’, applicants and drug candidates
2. initiative background and repurposing challenges
3. considerations on regulator involvement—role of the regulator, regulatory aims of repurposing initiatives
4. experiences & future perspectives—impact and results, practical solutions, recommendations

The complete interview guide is included in the Supplementary material.

### 2.3 Analysis

After obtaining interviewees’ written informed consent, the interviews were recorded and transcribed verbatim, or responses were obtained in writing. Thematic analysis according to Braun and Clarke (21) was conducted using NVIVO20 software, including mixed inductive and deductive coding based on the interview guide.

## 3 Results

**Figure 1:**
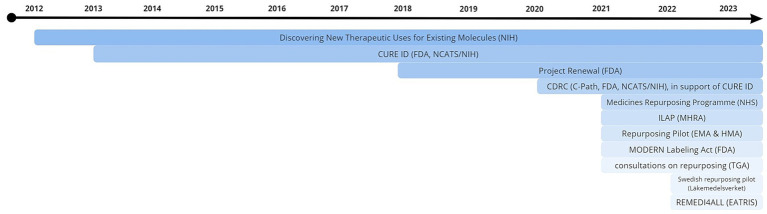
Timeline of current repurposing initiatives. The majority (*n* = 7) have been running for two years or less. Source: literature review as part of the current study.

Of the selected initiatives (*n* = 10), eight were included in the final analysis. Two organizations and initiatives were not included due to an inability to participate in the required timeframe (*n* = 1) or lack of response (*n* = 1). For one project, two representatives were interviewed on separate occasions, bringing the total number of interviews conducted to nine. An overview of participating agencies/institutions is shown in Table 1. A timeline of current initiatives, of which the majority (*n* = 7) have been running for two years or less, can be found in Figure 1. Respondents’ professional backgrounds ranged from regulatory affairs officer, director of national research centers, to executive directors of public private partnerships. Interviews were held individually, in-person (*n* = 1), remotely via video conferencing platform (*n* = 7), or by obtaining written responses to the interview questions (*n* = 1). A comprehensive overview of results is included in Table 2.

**Table 1:** Overview of invited interview candidates.

region	Organization	Initiative
Europe	EMA & the Heads of Medicines Agencies	Repurposing of Authorised Medicines (EU, 2021)
Europe	EATRIS, MEB, ZonMw ao	REMEDI4ALL (EU, 2022)
Sweden	Läkemedelsverket	Swedish pilot for repurposing (Sweden, 2022)
United Kingdom	Consortium of UK Health Agencies, MHRA	Innovative Licensing and Access Pathway (UK, 2021)
United Kingdom	NHS, supported by multiple agencies	Medicines Repurposing Programme (England, 2021)
United States	C-Path, FDA and NCATS/NIH	CURE Drug Repurposing Collaboratory (US, 2020), CURE ID (US, 2013)
United States	FDA	MODERN Labeling Act (US, 2021)
United States	FDA	Project Renewal (US, 2018)
United States	National Institutes of Health	Discovering New Therapeutic Uses for Existing Molecules (US, 2012)
Australia	Therapeutic Goods Administration	Currently exploring regulatory and policy reforms

As identified by region, organization, and initiative. Source: literature review as part of the current study. EATRIS, European Advanced Translational Research Infrastructure in Medicine; EMA, European Medicines Agency; FDA, Food and Drug Administration; MEB, Medicines Evaluation Board; MHRA, Medicines and Healthcare products Regulatory Agency; NCATS, National Center for Advancing Translational Sciences; NHS, National Health Service; NIH, National Institutes of Health.

**Table 2:** Overview of interview results: key considerations and exemplary quotes.

Main theme	Subthemes	Key considerations	Exemplary quotes	Number of respondents making similar statements
Initiative characteristics	Aims	Most initiatives aim to provide education, facilitate collaboration, and optimize the overall repurposing process.	We add value by increasing the efficiency of the system, the current understanding of the drug development progress. […] And reducing the systemic risk by bringing in the right people at the right time.	9	Some initiatives do not specifically target repurposing projects, but accept them as part of a broader scope.	We wanted to try to facilitate the interaction with the regulator in a way that had not been done before.	2
	Defining repurposing	The exact definition of ‘repurposing’ remains debatable.	Even within regulatory agencies, there may be different definitions of repurposing in use. Sometimes we may be talking about repurposing in the same context, but using a different term.	9	Most initiatives purposefully use a broad definition in order to attract a variety of applicants.	We take the broadest definition we can.	7
	Applicant candidates	While most initiatives employ a broad scope when selecting applicants, the majority targets academia, not-for-profit, or small companies.	The main feedback that we got was, instead of having quite narrow criteria, to broaden the definition, the scope.	5	We pick up those projects where there’s very limited commercial incentive for a company to take the lead.	5
	Drug candidates	While most initiatives employ a broad scope when selecting drug candidates, the majority targets off-patent, marketed drugs.The potential of shelved drugs is frequently considered.	We just do off-patent to make sure there is no infringement [on patent rights or exclusivity], because we do not have the resources or capability to deal with that.	3	I think repurposing marketed drugs is a nut we need to crack, because there are a lot [of diseases] that we are not treating that we could be treating.	4	Shelved drugs are a huge untapped area that would be worthwhile to explore	4
	Initiative’s background & repurposing challenges	Lack of regulatory expertise	Non-industry repurposing projects suffer from a knowledge gap regarding regulatory issues.	Navigating the regulatory or access system can be really challenging, many academic and not-for-profits are not very well resourced in terms of their capabilities.	9		We still have a huge mismatch between an academic’s understanding of the regulatory process, and the fact that they are actually working in a regulatory environment.	9		They do not even understand what types of questions they should ask the regulator.	7
		Lack of downstream perspective	Non-industry developers struggle to align and optimize their efforts along the drug development pathway, e.g., in clinical trial design.	Studies are potentially wasted because they are not designed in a way that could support a change to the marketing authorization.	8		Academics think in terms of publications.	8
		Lack of financial incentive	Industry is generally reluctant to engage in most repurposing projects due to the lack of a business case.	For-profit companies will look at it from a business perspective, it’s what they are there for. If they do not see commercial value, they are not doing it.	9	Once you are about to approach that generic time frame, [companies] start losing interest.	4	
			How do you convince a large pharmaceutical company to give you access to a drug that’s making billions a year in a rare disease, when all they see is risk?	4
Lack of awareness of pitfalls and opportunities		Non-industry developers tend to underestimate the complexities of repurposing.	There’s a lack of institutional support to understand what repurposing really entails.	5 3	The added value of regulatory involvement is often unclear.	There’s a lack of awareness, and perhaps a lack of appreciation for the regulatory system.	3
Lack of collaboration		Multi-stakeholder engagement remains challenging.	Companies do not work together, payors do not work together.	4	Repurposing efforts are often fragmented.	All these different aspects need to be considered. This is not an individual undertaking, there has to be a context or platform where we can start having these discussions.	4
Lack of data access		Data monopolies hamper non-industry repurposing efforts.	There’s a vast amount of information that does not seem to be readily accessible to those wanting to do repurposing. If you rely solely on published data, that’s actually just scratching the surface in terms of information.	5
Lack of sponsor		Misalignment of legal and regulatory incentives complicates finding sponsors willing to take on the responsibilities associated with repurposing efforts.	When there is not a pharmaceutical company taking the lead, it’s quite hard for a researcher to take all those steps [e.g. licensing, guideline development] themselves.	5	With lack of a sponsor comes a lack of project coordination.	We hear a lot of concerns from pharmaceutical companies about cheap alternatives appearing on the market, about off-label use suddenly becoming on-label. Companies feel like they are losing control, because they are used to being the only ones in control of drug development.	2	Without some sort of partnership or support, there would be reluctance from companies even just because of the effort and workload involved.	4
Lack of legislative and regulatory capabilities		It is challenging for regulators to structurally support repurposing within the current legal and regulatory framework.	Oftentimes, regulators are handcuffed. There’s a limited amount that they can do at this point.	4
Regulator efforts		Regulators can facilitate repurposing through early involvement, education, lowering the threshold for regulatory involvement, fostering collaboration, and building awareness.	We [regulators] are the gatekeepers, but we want to talk to you, and hear about your projects early.	8	Engaging with the regulator is really important very early in a repurposing program, to understand what the objectives are right from the beginning.	8	We wanted to facilitate the interaction with the regulator by having something quite visible, to be able to signpost to academics and not-for-profits and potentially companies, that this is a pathway to provide you with additional regulatory support.	6	
			We have to be different from what we ordinarily are, when we meet pharmaceutical companies. Not that we are hostile, we are always friendly, but it’s another way of taking on a project: creating a friendly, welcoming, open atmosphere to ask questions.	2	There’s a real possibility that regulators may be in the best position to negotiate with drug owners. [e.g. when it comes to incentivizing data access, or MA variations]	1	It’s important for researchers to feel that someone is supporting them, something is happening where they can share their thoughts and ideas with colleagues dealing with the same challenges, to have them support each other and make them aware. Regulatory authorities can facilitate such a network.	4
Repurposing end goal		While patient access is the main focus for all initiatives, bringing new indications on label remains the golden standard for many repurposing efforts, for safety, efficacy, as well as financial reasons.	Having the patient right at the forefront of your mind when you are considering your repurposing program is absolutely crucial.	9	The goal is to get drugs to patients, as widely as possible. On-label where possible, off-label only if really necessary.	7	Market authorization is really key, so that prescribers can prescribe with confidence, with a greater possibility for reimbursement.	7
experiences & future perspectives		impact & results	An increasing amount of interest in repurposing seems apparent.Several initiatives have seen concrete successes already, but the majority of initiatives have recently started.	We know there’s this appetite for [repurposing].	6	General stakeholder feedback is very positive all across the board.	9
		Possible practical solutions	Several practical solutions to current repurposing barriers are suggested, including legislative changes (e.g. the European pharmaceutical legislation revision), regulatory changes (e.g., transferable exclusivity), funding system changes (e.g., a pooled innovation fund, using existing public funding mechanisms), drug reformulation, and diversification of scientific advice.	Legislative changes in Europe could reverberate around the world.	8		Things like transferable exclusivity are 100% in the regulator’s wheelhouse, along with legislators.	1	We’ve had ‘meet the regulator’, kind of like speed dating, an informal way of scientific advice where companies and academia can ask all sorts of questions in a short session.	2
		Recommendations	Increased (international) stakeholder engagement is recommended.	How do you know you have a drinking problem? First of all, you have to admit that you have a problem, right? We have to admit there’s a problem in repurposing. Let us come together and talk about it from a global perspective.	5	Harmonization of efforts is favored.	We want to make it where it’s like, no, we are going to do this together guys, because this is it: you are all going to benefit, and the benefit is going to be worth overcoming the barriers of working together.	7	Legislative changes may allow for more long-term, sustainable changes to the current repurposing framework.	The intention is to bring initiatives together, so that we can share what we are doing and learn from each other, and see whether in the long-term we can move towards more formal data sharing agreements, trials and regulatory dossiers collaboration, *etcetera*.	5

Main themes and subthemes are shown, as based on the interview guide, along with key considerations, exemplary quotes, and the number of individual respondents who made similar statements (#). A total of 9 respondents were interviewed, representing 8 projects. Source: Authors’ analysis of data from the semi-structured interviews, conducted in summer 2023, with international regulators and leaders of publicly-funded repurposing efforts with regulator involvement. MA, marketing authorization.

### 3.1 Initiative characteristics

Respondents for all initiatives (*n* = 9) indicated that their projects aim to achieve one or more of the following: to provide education on drug repurposing, facilitate collaboration on repurposing projects, and/or optimize the overall repurposing process. The majority (*n* = 8) purposefully employ a broad conceptualization of repurposing, to attract as widespread a pool of (drug) candidates as possible, and to lower the threshold for involvement of drug developers. While most initiatives are open to a wide range of participants (*n* = 5), the majority target academia, not-for-profit organizations, or small companies (*n* = 5). Five initiatives also specifically target off-patent, marketed drugs. Two initiatives do not specifically target repurposing projects but include them as part of a broader program. The potential of shelved compounds (discontinued or put on hold during development) is also frequently considered (*n* = 4). All respondents (*n* = 9) agreed that the definition of repurposing remains hotly debated (e.g., the difference between repurposing and repositioning). These issues were perceived by four respondents to complicate repurposing efforts considerably, as certain examples may not even be acknowledged as ‘repurposing’ (quote 1).

“Even within regulatory agencies, there may be different definitions of repurposing in use. Sometimes we may be talking about repurposing in the same context but using a different term.” Quote 1.

### 3.2 Initiative background and repurposing challenges

The most commonly perceived barriers to repurposing were:

1. lack of familiarity with regulatory requirements (as illustrated by quote 2): repurposing projects conducted by non-traditional developers (entities not primarily associated with the pharmaceutical industry, such as academia) suffer from a knowledge gap by these developers of regulatory issues (e.g., they do not know what data is needed, or which questions to ask regulators) (*n* = 9). This also results in a limited downstream drug development perspective: non-traditional developers struggle to align and optimize their efforts along the drug development pathway (e.g., clinical trials are not set up in way that support MA) (*n* = 8). The knowledge gap may also lead to inadequate awareness of pitfalls and opportunities (as illustrated by quote 3): non-traditional drug developers tend to underestimate the complexities of repurposing and the added value of regulatory involvement can be unclear (*n* = 5)
2. insufficient financial incentives: industry is generally reluctant to engage in repurposing projects due to the lack of a business case and limited perceived return on investment (*n* = 9)
3. poor collaboration: multi-stakeholder engagement remains challenging, repurposing efforts are often fragmented and operate in siloes (*n* = 4)

“We still have a huge mismatch between an academic’s understanding of the regulatory process, and the fact that they are actually working in a regulatory environment.” Quote 2.

“There’s a lack of awareness, and perhaps a lack of appreciation for the regulatory system.” Quote 3.

### 3.3 Regulatory relevance

While patient access is the main focus for all initiatives (*n* = 8) (quote 4), label extension remains the gold standard (*n* = 6), to demonstrate safety and efficacy of the product, as well as for financial reasons. While label extension may be the preferred outcome, six lead respondents (*n* = 6) regarded widespread off-label use as ‘successful repurposing’, due to the challenges they perceived in obtaining regulatory approval for new indications in some cases. Regulatory approval offers advantages such as a pharmacovigilance framework, the ability of prescribers to prescribe with confidence, and equal access to evidence-based treatment. Reimbursement may also be dependent on whether an indication is included in the label. Ways for regulators to facilitate repurposing include early involvement (*n* = 8), education (*n* = 8), making regulators readily approachable for feedback on repurposing projects (*n* = 6), fostering collaboration (*n* = 8), and building awareness among drug developers (including atypical ones) about the regulatory support available and its added value (*n* = 4).

“The goal is to get drugs to patients, as widely as possible. On-label where possible, off-label only if really necessary.” Quote 4.

### 3.4 Experiences and future perspectives

The last several years have witnessed increasing interest in drug repurposing (*n* = 6). Seven initiatives have seen concrete success, from securing data packages from clinicians and industry to repurposed drugs making it into phase III trials, although six projects started relatively recently (Figure 1). How these successes are defined depends on the project specific aims, and do not necessarily have to include label extensions. Plans for impact analysis are in place for nearly all (*n* = 7). These include generation and publication of interim assessment reports based on stakeholder feedback, as well as analysis of drug prescription statistics. Several practical solutions were mentioned with regard to the initiation of a repurposing pilot. These include keeping proceedings as simple as possible to ensure efficiency (*n* = 2), maintaining a helicopter view of the entire process both up and downstream (*n* = 3) (e.g., design clinical trials based on a prospective change to the MA), and taking lessons from the COVID-19 pandemic, where sudden pressing unmet medical need enabled successes like the RECOVERY trial (*n* = 1). A rethinking of the role of the regulator was also suggested by four respondents: aiming to have a more informal approach towards non-traditional developers. Opportunities within the current regulatory and legal system can and should be utilized. Examples include incentivizing MAHs to change labels by creating financial incentives using publicly funded mechanisms to study repurposed drugs and have them labeled for their new indications (*n* = 3). More disruptive ideas were suggested by some (e.g., a pooled innovation fund focusing on payors (*n* = 1): if health insurers save money through repurposing, new repurposing projects could be funded by taking a percentage of those savings). Overall recommendations by respondents included increased (international) stakeholder engagement, which could be improved by actively engaging with and specifically addressing concerns raised by industry, (*n* = 4). Harmonization of repurposing endeavors was favored by seven respondents, for which they indicated that efforts are currently underway. Legislative changes may enable the current repurposing ecosystem to evolve, and the majority of interviewees (*n* = 7) were excited about the EU pharmaceutical legislation reform and repurposing’s prominence therein. Finally, a concern shared by most respondents (*n* = 5) is creating awareness that there is a problem in repurposing, underlining the need to start thinking in terms of practical solutions in order to move forward in a sustainable way (quote 5).

“How do you know you have a drinking problem? First of all, you have to admit that you have a problem, right? We have to admit there’s a problem in repurposing. Let us come together and talk about it from a global perspective.” Quote 5.

## 4 Discussion

In summary, this study found that the studied repurposing initiatives are in agreement regarding general aims and the preference for an inclusive approach. A lack of familiarity with regulatory requirements and financial incentives are widely recognized to be two of the main barriers to successful repurposing for non-traditional drug developers. As regulatory approval remains the gold standard, the relevance of regulator involvement is acknowledged across the board. Nevertheless, fundamental differences remain, most notably when it comes to the definition of repurposing.

Repurposing seems to have outgrown the initial phase of turmoil and underappreciation that characterized its beginnings as a topic of interest, as the community has developed a variety of concrete initiatives with a shared recognition of necessity, potential, and goals. Questions of definition and who will take ownership of repurposing ventures remain to be addressed. The lack of consensus on the exact definition of repurposing may hamper repurposing harmonization efforts (2). Some respondents were reluctant to even refer to their endeavors as repurposing. While this lack of consistency complicates matters, it does not seem to keep efforts from moving forward. Utilization of a broad definition of repurposing may be advantageous, as it opens programs to a wider variety of stakeholders. Moreover, it remains unclear who (government, not-for-profit, patient organizations, etc.) will lead repurposing ventures, particularly when drugs are off-patent. While the initiatives included in this study are all led by regulators or publicly-funded entities, multi-stakeholder involvement is considered essential.

Stakeholders share a recognition of repurposing’s necessity, potential, and goals, as well as general agreement on the primary barriers to its success. This could offer opportunities for collaboration and harmonization and confirms the progress the repurposing ecosystem has experienced since its inception. The knowledge gap in regulatory expertise for non-traditional repurposing programs is being addressed by initiatives like STARS and the IRDiRC Task Force but remains a challenge (17, 22, 23). In limited cases regulators are incentivizing industry to update labels using existing mechanisms within their regulatory framework. Though a shared vision for the general aims of an ideal repurposing ecosystem has developed, this agreement has not yet resulted in demonstration of large-scale successful repurposing projects. However, current, newly-initiated programs show the potential of repurposing when diverse stakeholders work together, and should be closely watched. REPO4EU is an example of another multi-stakeholder initiative, in which regulators are not (yet) directly involved, which aims to develop an online platform for drug repurposing. Time will tell if these and other initiatives result in concrete successes and scalable solutions. A very recent study of Liddicoat et al. (24) proposes five criteria that could be used to determine the success of these initiatives: the number of authorizations granted; their clinical impact; the number of patients treated in accordance with the new use; savings in public healthcare; and reductions in off-label use, especially if the earlier use was based on inadequate data. Exciting accomplishments such as the recent licensing of breast cancer drug anastrozole as preventive treatment by the Medicines and Healthcare products Regulatory Agency in the United Kingdom, supported by the NHS Medicines Repurposing Programme, are promising (25).

Regulators can help address these challenges by reassessing their role in the repurposing ecosystem. As respondents indicate, different regulators are already seeking to reshape their responsibilities in repurposing in different ways: from organizing accessible education sessions tailored to non-traditional drug developers to proactive involvement with academia. Regulators can help maintain consistently high research standards in drug trials by providing scientific advice, for example. To what extent this type of education should fall under the regulator’s scope or might be better suited to be taken up by academia themselves, is a topic of discussion. For regulators, while maintaining objectivity remains key, a shift to a more proactive, collaborative approach involving increased outreach to non-traditional drug developers seems apparent and necessary (especially within the rare disease realm). This reassessment could benefit from a two-tiered approach, one focusing on practical measures, the other on legislative changes. Practical actions may include the (international) upscaling of existing initiatives, early and accessible regulatory involvement in repurposing projects by providing clear and consistent guidance, and increased collaboration with, for instance, publicly funded entities. This is where legislative changes may also be helpful, as they would enable governments and non-traditional drug developers to take on responsibilities that have traditionally lain with industry. An example of a similar, successful approach is the Best Pharmaceuticals for Children Act (BPCA), that prioritizes pediatric therapeutic needs by bringing these on-label. In a similar vein, the newly proposed EU pharmaceutical legislation reforms mention repurposing as well (26). The draft text states that, in case of substantive evidence for a new therapeutic indication expected to fulfill an unmet medical need, “…marketing authorisation holders of the medicinal products concerned shall submit a variation to update the product information with the new therapeutic indication.” No matter the practical interpretation, it is encouraging to see that, on an advanced level, repurposing and its potential are firmly on the agenda.

Limitations of this study include the fact that despite stating various practical suggestions for advancing repurposing from a regulatory perspective, this study did not dive further into their feasibility. This study confirms that it remains challenging to get an easily attainable overview of available repurposing resources, including regulatory and publicly-funded initiatives, for researchers and developers alike. Despite these initial difficulties, active engagement with the international regulatory network (e.g., at C-Path’s CURE Drug Repurposing Collaboratory annual meeting in Washington DC in April 2023) enabled the establishment of the comprehensive overview of these projects presented here. Future research could focus on perspectives of other stakeholders (industry, academics, patients) in the current repurposing context, the use of innovative methods (such as artificial intelligence) in repurposing, assessment of the results of the repurposing initiatives currently underway (including international trend analysis), as well as the possible implications of the proposed EU pharmaceutical legislation reforms.

## 5 Conclusion

This study shows that, based on current regulator and publicly-funded initiatives to advance repurposing, the concept has entered a new phase, seeing a variety of concrete initiatives with a shared recognition of necessity, potential, and goals, as well as the identification of a lack of familiarity with regulatory requirements and financial incentives as two main barriers to its success. Within the confines of the current repurposing ecosystem, regulators may be able to provide educational support and guidance to organizations focused on drug repurposing for unmet medical needs, whilst maintaining their traditional and essential objectivity. Legislative initiatives may also help to further the use of drug repurposing for unmet medical needs.

## Data availability statement

The original contributions presented in the study are included in the article/Supplementary material, further inquiries can be directed to the corresponding author.

## Author contributions

ES: Formal analysis, Investigation, Methodology, Writing – original draft, Writing – review & editing. AM-T: Writing – review & editing. AP: Conceptualization, Formal analysis, Methodology, Writing – review & editing.

## Glossary

ADMET: absorption, distribution, metabolism, excretion, toxicity
API: active pharmaceutical ingredient
BPCA: Best Pharmaceuticals for Children Act
EATRIS: European Advanced Translational Research Infrastructure in Medicine
EMA: European Medicines Agency
EU: European Union
FDA: United States Food and Drug Administration
ILAP: Innovative Licensing and Access Pathway
IRDiRC: International Rare Diseases Research Consortium
MA: marketing authorization
MAH: marketing authorization holder
MEB: Dutch Medicines Evaluation Board
MHRA: Medicines and Healthcare products Regulatory Agency
NHS: National Health Service
NCATS: National Center for Advancing Translational Sciences
NIH: National Institutes of Health
R&D: research and development
RUF: Reagan-Udall Foundation
TGA: Therapeutic Goods Administration
