# Supplementary material for: International regulatory and publicly-funded initiatives to advance drug repurposing
Source: Front Med (Lausanne). 2024 May 31;11:1387517. doi: 10.3389/fmed.2024.1387517 (PMC11177848; doi:10.3389/fmed.2024.1387517)
Supplement: Supplementary file 1 [file Table_1.docx]

Supplementary Material

**Supplementary Table 1.** **Comprehensive interview guide used in all interviews (n=9).** Interview sub questions were devised to explore the included topics in a structured manner, as based on the literature review. In bold are the main questions per topic. Bullet points in blue are sub questions. Cursive and blue text are examples of quotes that might be used in the interview.

| Topic | Questions |
| --- | --- |
| Introduction | Could you introduce yourself briefly?  How would you describe/define drug repurposing?  How do your responsibilities involve drug repurposing? |
| Defining aspects of repurposing initiatives | How is ‘repurposing’ defined within this initiative?   - How does this definition relate to the initiative’s workings? - Is the initiative tailored to involve approved / off patent / … drugs only?   *Eg: As we think about drug repurposing, we can think of examples like…*   - For whom (industry, academia, non-for-profit) + what (therapeutic areas) is the initiative intended? |
| Background & challenges | How does this initiative aim to mitigate challenges surrounding repurposing?   - What did you notice to be a bottleneck in repurposing efforts? - What prompted the creation of this initiative?   *Eg: example of EMA pilot: A lack of regulatory knowledge among academia and non-for-profit developers may hamper repurposing efforts. Hence, the EMA pilot offers regulatory support especially for these target groups.* |
| Regulatory relevance | How do you view the role of the regulator in repurposing?   - What should be the end goal of repurposing?   *Eg: Regulatory approval / uptake into professional guidelines / ….*   - What should regulators be doing for repurposing in unmet needs?   *Eg: example of government taking this approach (in pediatrics): BPCA*.   - What are ways for regulators to facilitate repurposing for unmet needs (without financial incentive)? - How to approach this in practical terms from a regulatory perspective? |
| Experiences & future perspectives | What are lessons you/your organization have learned from your current repurposing initiative(s)?   - Has the impact of this initiative been analyzed? - What would you do differently in the future? |
| Ending | Any additional/concluding perspectives, opinions, arguments, or notions that you would like to share?  Can I contact you after the interview to discuss new or missing information? |
